# Immune mechanism of gut microbiota and its metabolites in the occurrence and development of cardiovascular diseases

**DOI:** 10.3389/fmicb.2022.1034537

**Published:** 2022-12-14

**Authors:** Jing Lu, Xiao Jin, Shengjie Yang, Yujuan Li, Xinyue Wang, Min Wu

**Affiliations:** Guang’an Men Hospital, China Academy of Chinese Medical Science, Beijing, China

**Keywords:** cardiovascular diseases, immune mechanism, intestinal microbiota, metabolites, gut microbiota regulation

## Abstract

The risk of cardiovascular disease (CVD) is associated with unusual changes in the human gut microbiota, most commonly coronary atherosclerotic heart disease, hypertension, and heart failure. Immune mechanisms maintain a dynamic balance between the gut microbiota and the host immune system. When one side changes and the balance is disrupted, different degrees of damage are inflicted on the host and a diseased state gradually develops over time. This review summarizes the immune mechanism of the gut microbiota and its metabolites in the occurrence of common CVDs, discusses the relationship between gut-heart axis dysfunction and the progression of CVD, and lists the currently effective methods of regulating the gut microbiota for the treatment of CVDs.

## Introduction

Cardiovascular diseases (CVDs) are the most dangerous cause of unnatural human death and mainly include coronary heart disease, hypertension, and heart failure. In recent years, the prevalence and mortality rate of CVDs worldwide have increased. In 2019, CVDs caused 6.2 million deaths between the ages of 30 and 70 years (Roth et al., 2020). Many factors affect CVDs development, including genetic inheritance, age, sex, diet, and lifestyle (Sniderman and Furberg, 2008; Regitz-Zagrosek and Kararigas, 2017; Badimon et al., 2019; Hosen et al., 2020). Gut microbiota and their metabolites have a significant impact on CVD. The gut microbiota is responsible for energy absorption, substance metabolism, and immune responses, and the stability and elasticity of their basic ecological characteristics have a significant impact on the human internal environment (Fassarella et al., 2021). The gut microbiota changes in composition and quantity, and their metabolites change with it, affecting the metabolism, immune response, and intestinal barrier function of the host (Rajendiran et al., 2021), which is reflected in the interaction with the host innate and the adaptive immune systems. In CVDs, the relationship between the gut microbiota and the host immune system should not be overlooked. This review focuses on the immune mechanism and explores this process in detail, hoping to find a new therapy to modulate intestinal bacteria specifically for CVDs.

## CVDs and the gut microbiota

### Formation and development of the gut microbiota

The human gut microbiota is a large biological community, it varies from person to person just like the unique gene pool in every human body, and these bacteria have co-evolved with humans (Turnbaugh et al., 2010; Lepage, 2017). The gut microbiota is present in the neonatal period and changes dynamically over time; however, in the placenta, amniotic fluid, and meconium, some researchers have found bacteria such as *Proteobacteria*, *Bacteroidetes*, *and* Firmicutes (Perez-Munoz et al., 2017; Liu et al., 2022). The gut microbiota of babies born vaginally changes dramatically within several weeks, a portion of the microbiota is eliminated or replaced, and the main microbiota changes from facultative anaerobic to strict anaerobic bacteria, such as *Bifidobacterium* (Ferretti et al., 2018). After that, the composition of the microbiota is affected by factors such as feeding style, family environment, and antibiotic use (Bokulich et al., 2016), after 7–12 years old, it will gradually approach that of adults (Hollister et al., 2015), and remain relatively stable thereafter, laying a good foundation for the maturity of the immune system and improving gastrointestinal mucosal barrier function (Mendez et al., 2021). In elderly people, the abundance of intestinal Bacteroides and other bacteria is decreased, moreover, there is a decrease in intestinal mucosal barrier function, with significantly reduced beneficial bacteria levels compared to those in young individuals. (Odamaki et al., 2016). However, there is one exception, the gut microbiota of long-living people has a higher abundance of Bacteroidetes phylum and exerts beneficial functions (Zhang S. et al., 2021). Bacteroides are involved in the metabolism of bile acids and short-chain fatty acids (SCFAs) and have a protective effect against atherosclerosis (Winston and Theriot, 2020). An increased Firmicutes (F) and Bacteroides (B) ratio (F/B) is associated with the decreased production of SCFAs (Bifari et al., 2017), which increases the risk of CVDs. The gut microbiota is closely related to the occurrence of CVDs, and the diversity of intestinal flora in different CVD states also shows subtle differences, producing different bacterial metabolites, which in turn regulate CVDs with inflammation and immune mechanisms (Table 1).

**Table 1:** Changes and characteristics of the gut microbiota in patients with three common CVDs.

Cardiovascular diseases	Patient population	Main findings/Outcomes	Mechanism	References
Atherosclerosis	70 with CAD, and 98 Ctrls	The relative abundance of Firmicutes, Bacteroidetes decreased, while *Proteobacteria* and *Actinobacteria* increased in the CAD group, *Escherichia-Shigella, Lactobacillus*, and *Enterococcus* were found to be significantly enriched in the CAD group.	Inflammation caused by disturbances in the gut microbiota accelerate the progression of coronary heart disease.	Zhu et al. (2018)
	39 CAD patients，and 50 healthy volunteers	The *Lactobacillales* was increased, whereas the phylum Bacteroidetes (*the genera Bacteroides*, *Prevotella*) was decreased in the CAD group.	*Bacteroides fragilis* can promote regulatory T-cell function, regulating adaptive immune.	Emoto et al. (2016)
	Of the 108 MZ twins, 14 pairs discordant for carotid intima-media thickness (IMT) were selected to undergo a stool sample analysis	The group with high IMT values had low microbiota diversity, Firmicutes/Bacteroidetes ratio was greater; the Firmicutes had higher abundance, whereas that of Prevotellaceae was lower. Normal carotid IMT values were associated with a substantially higher fraction of Prevotellaceae.	The carotid-femoral pulse wave velocity (PWV) was negatively correlated with gut microbiome alpha diversity. The specific mechanism awaits further study.	Szabo et al. (2021)
	41 controls, 56 subjects with pHTN and 99 with primary HTN	The microbiome characteristic in pre-hypertension group was quite similar to that in hypertension, genera such as *Prevotella* and *Klebsiella* are overepresented in individuals with pHTN or HTN, a reduction of *Faecalibacterium, Oscillibacter, Roseburia, Bifidobacterium, Coprococcus* and *Butyrivibrio.*	Prevotella may trigger the inflammatory response; over production of LPS by gut microbiota seems to be directly linked to HTN development.	Li et al. (2017)
Hypertension	4,672 subjects (mean age 49.8 ± 11.7 years, 52% women) from six different ethnic groups participating in the HELIUS study	The abundance of *Roseburia* spp., *Clostridium sensu stricto* spp., *Roseburia hominis*, *Romboutsia* spp., *Streptococcus* spp., and *Ruminococcaceae NK4A214* spp. was negatively associated with both SBP and DBP.	SCFA-producing microbes are associated with lower BP.	Verhaar et al. (2020)
	SHR models; two rat models of hypertension and a small cohort of patients was used for bacterial genomic analysis	Lactate-producing bacteria (*Streptococcus* and *Turicibacter)* were in higher quantities in the SHR, the F/B ratio was increased, a dysfunction in both acetogenic and butyrogenic capabilities.	HTN-associated dysbiosis is characterized as an accumulation of lactate-producing bacteria and a reduction of acetate and butyrate producers.	Yang et al. (2015)
	213 pregnant women, 11 women with pre-eclampsia (DPE), 202 controls	Women with DPE had significantly lower (alpha) diversity in their gut microbiota, and the SCFAs-producers, such as *Coprococcus*, Roseburia, *Lachnospira*, *Butyricimonas*, *Unclassified* Clostridiaceae, and *Unclassified* Clostridiales are reduced compared to the control group.	Reduced numbers of butyrate-producing bacteria in the gut microbiota induces lower circulating butyrate levels, influence SBP during early pregnancy.	Altemani et al. (2021)
	20 with HF due to frequent etiologies like ICMP and DCM, 20 healthy control subjects	*Coriobacteriaceae*, *Erysipelotrichaceae* and *Ruminococcaceae*, *Blautia*, *Collinsella*, uncl. *Erysipelotrichaceae*, and uncl. *Ruminococcaceae* showed a significant decrease, *Escherichia/Shigella* were enriched in HF cases. The pattern of depleted genera *Blautia* and *Collinsella* seems to be HF specific.	Intestinal epithelial dysfunction led to increasing permeability, possibly induced a bacterial shift and given rise to systemic inflammation.	Luedde et al. (2017)
Heart failure	60 well-nourished patients in stable condition with CHF; 20 matched healthy control subjects	Compared with normal control subjects, the entire CHF population had massive quantities of pathogenic bacteria and *Candida*, such as *Campylobacter, Shigella,* Salmonella*, Yersinia enterocolitica,* and *Candida* species.	Antimicrobial, hypoxia and acid/base disturbance, bowel ischemia, gastrointestinal dysmotility et al. may induce overgrowth of pathogenic bacteria and translocation.	Pasini et al. (2016)
	22 patients admitted for HF and 11 control subjects without a history of HF	Compared with control subjects, the phylum *Actinobacteria* and *Bifiodobacterium* was enriched whereas *Megamonas* was depleted, and plasma concentration of trimethylamine N-oxide (TMAO) was increased in HF patients; compared with the compensated HF patients, the decompensated HF had more abundant *Escherichia/Shigella* in the same patient.	An abundance of *Escherichia/Shigella cluster* means more TMA lyase (CutC/D) gene and higher circulating TMAO levels, implying certain bacteria harboring TMA lyases may enrich in the decompensated phase of HF.	Hayashi et al. (2018)

CAD, coronary artery disease; HTN, hypertension; pHTN, pre-hypertension; HELIUS, Healthy Life In an Urban Setting; SBP, systolic blood pressure; DBP, diastolic blood pressure; SHR, spontaneously hypertensive rat; HF, heart failure; ICMP, ischemic cardiomyopathy; DCM, dilated cardiomyopathy; CHF, chronic heart failure; DPE, pre-eclampsia.

### Coronary atherosclerosis and the gut microbiota

Atherosclerosis is the pathological basis of coronary heart disease. Compared to healthy volunteers, the order *Lactobacillales* increased, whereas the phylum Bacteroidetes was significantly decreased in patients with coronary artery disease (CAD) (Yamashita et al., 2016). The gut microbiota play an important role in atherosclerosis development, there are many pro-inflammatory microbiota in the gut of atherosclerotic patients, such as *Collinsella*, *Escherichia coli*, *Klebsiella* spp., *Enterobacter aerogenes*, *Streptococcus spp*., and *Lactobacillus salivarius,* while only a few fermentative microbiota, such as *Bacteroides spp*., *Prevotella copri*, and *Alistipes shahii* (Table 1). Patients with atherosclerosis have a higher abundance of the genus *Collinsella*, which can activate neutrophils, promote the release of inflammatory factors, and aggravate the inflammatory response (Karlsson et al., 2012). In contrast, they have lower levels of fermentative microbiota, making SCFAs generated by fiber fermentation decrease (Liu et al., 2019; Zhou et al., 2020), SCFAs can reduce plaque formation by anti-inflammatory, immunomodulatory, and enhanced cholesterol efflux (Du et al., 2020; Piccioni et al., 2021), the decreased production of SCFAs undoubtedly promote the progression of atherosclerosis. Additionally, a choline-rich diet led to Trimethylamine N-oxide (TMAO) levels increasing, under the action of gut microbiota, the choline, phosphatidylcholine, and l-carnitine are metabolized into trimethylamine (TMA), which is converted to TMAO in the liver, the high level of TMAO in the blood becomes the risk factor of atherosclerosis (Wang et al., 2011; Ma and Li, 2018).

### Hypertension and the gut microbiota

Hypertension is a risk factor for CVD morbidity and mortality. According to 16S ribosomal RNA sequencing, the gut microbiota of patients with hypertension showed changes in richness, decreased diversity, and an increased F/B ratio (Avery et al., 2021). For example, *Christensenella* is more abundant in individuals with hypertension and is negatively associated with systolic blood pressure. Changes in diastolic blood pressure can also change the abundance of *Christensenella* and *Olsenella* (Dan et al., 2019). Disorders in the gut microbiota may also limit the production of vitamin D by specific bacteria in the gut, which affects blood pressure (Zuo et al., 2019). In addition, hypertension is related to the reduction in bacteria that produce acetate and butyrate in the gut microbiota. Animal experiments have shown that acetate and butyrate treatment reduce blood pressure by affecting renin release and blood pressure regulation through G protein-coupled receptors, whereas there is no significant antihypertensive effect in rats with normal blood pressure (Kim et al., 2018). Gut microbiota is closely associated with salt-sensitive hypertension, and a high-salt diet increases intestinal colonization of phylum Firmicutes and increases inflammation of the intestine and blood vessels, consequently leading to changes in blood pressure (Elijovich et al., 2020). In hypertensive patients, the phylum Firmicutes is significantly reduced, and an increase in Bacteroides indicates a correlation between Firmicutes (F) and Bacteroides (B) ratio and hypertension (Yang et al., 2015).

Hypertension increases intestinal permeability can be explained by several mechanisms, such as endothelial injury-induced inflammation, intestinal mucosal hypoperfusion, therefore, bacterial components, the lipopolysaccharides (LPS), can easily enter circulation by breaking the mucosal barrier (Li et al., 2020). This stimulates vascular toll-like receptors (TLRs), leading to low levels of chronic inflammation and exacerbation of hypertension (Santisteban et al., 2017; Robles-Vera et al., 2020). Moreover, changes in gut microbiota can activate the sympathetic nervous system and induce blood pressure fluctuations (Sharma et al., 2019). Plasma TMAO levels in hypertensive patients are positively correlated with blood pressure, which exacerbates angiotensin II (Ang II)-induced vasoconstriction by activating protein kinase R-like endoplasmic reticulum kinase (PERK) and its downstream reactive oxygen species/calmodulin-dependent protein kinase II/phospholipase C β3/Ca^2+^ (ROS/CaMKII/PLC β3/Ca^2+^) pathways (Jiang et al., 2021). This ultimately affects the blood pressure. In addition, the gut microbiota produces hormones and peptides that influence sodium absorption, such as gastrin, glucocorticoids, and glucagon-like peptide-1. Sodium (Na) plays an important role in the pathogenesis of hypertension. Based on these, blocking sodium absorption from the gut is becoming a new option for the regulation of blood pressure (Afsar et al., 2016).

### Heart failure and the gut microbiota

Patients with heart failure have impaired cardiac congestion or ejection capacity, resulting in inadequate perfusion of blood to internal organs, ischemic edema in the intestine, and increased permeability of intestinal epithelial cells, therefore, the gut microbiota and bacterial metabolites enter the circulatory system through a damaged barrier, inducing local and systemic inflammatory responses; this is the classic theory of “intestinal leakage” (Sandek et al., 2007; Mamic et al., 2021; Zhang Y. et al., 2021). A reduction in the healthy gut microbiota in patients with heart failure may promote the growth of pathogenic bacteria. In patients with heart failure, *Faecalibacterium prausnitzii* decrease and *Ruminococcus gnavus* increase are the basic characteristics (Cui et al., 2018), along with an increased abundance of several pathogenic bacteria and *Candida*, such as *Campylobacter, Shigella, Salmonella, Yersinia enterocolitica* (Pasini et al., 2016). In contrast, butyrate-producing bacteria is relatively reduced, butyrate level decreases, tumor necrosis factor-α (TNF-α)/interferon-γ (IFN-γ) upregulate claudin-2, inducing the intestinal immune barrier destruction, endotoxin LPS leakage (Huang et al., 2021). The gut microbiota of patients with myocardial infarction also changes significantly, with a decrease in the abundance of Pachychobacteria and an increase in the abundance of Bacteroides (Han et al., 2021).

## Role of gut microbiota and their metabolites in the immune mechanisms of CVD progression

**Figure 1:**
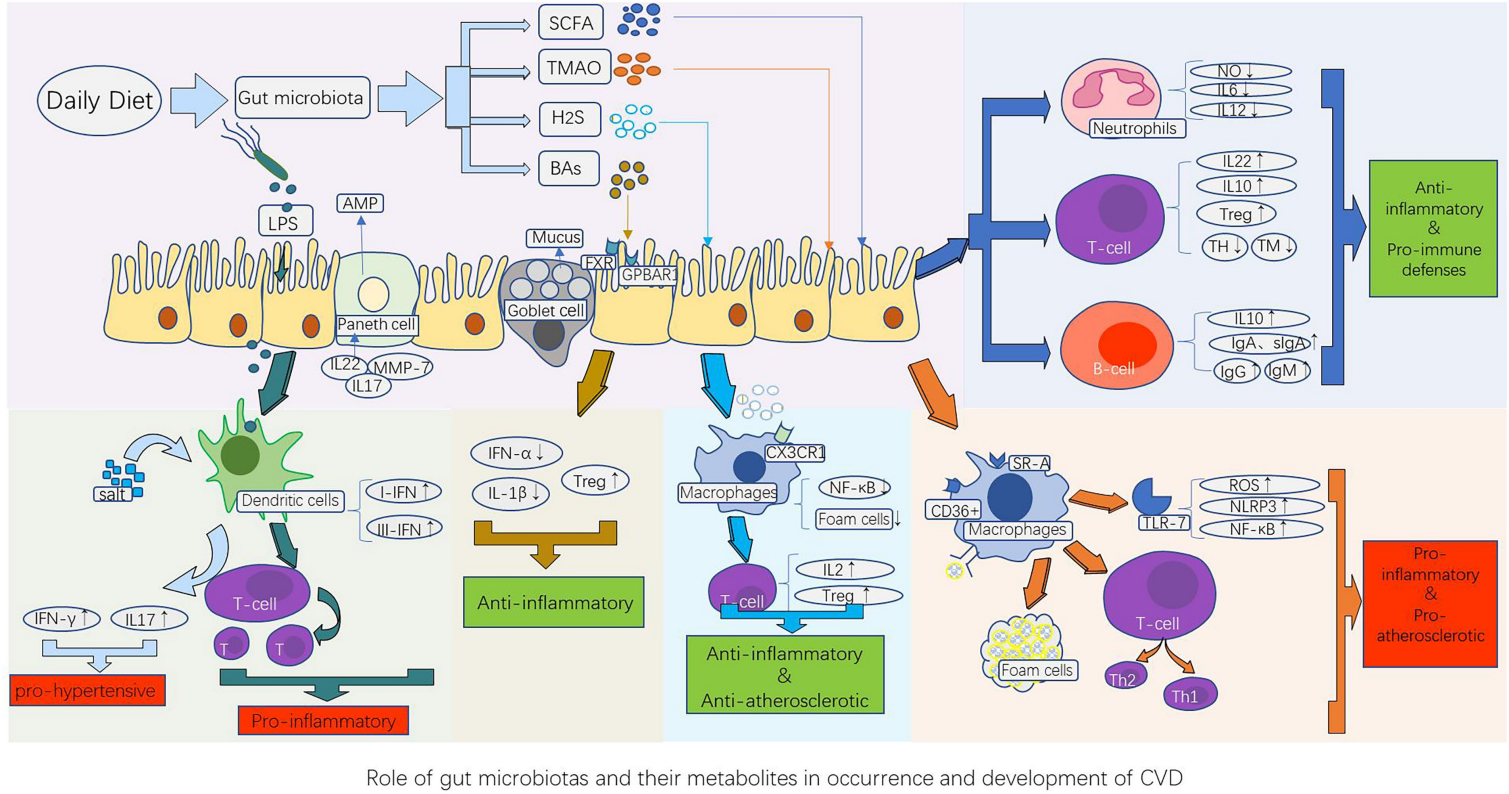
Role of gut microbiota and their metabolites in the occurrence and development of CVD. The gut microbiota component LPS, as well as their metabolites such as short-chain fatty acids (SCFAs), trimethylamine N-oxide (TMAO), bile acids (BAs), and hydrogen sulfide (H2S), stimulate the innate immune system (dendritic cells, macrophages, and neutrophils) and adaptive immune system (B cells and T cells), thereby causing the corresponding immune response of the host cardiovascular system. This produces a series of pathological changes by promoting or inhibiting the release of cytokines and inflammatory factors, such as atherosclerotic plaque formation, vasomotor dysfunction, and cardiomyocyte ischemia and necrosis, which influence the occurrence and development of CVDs. AMP, antimicrobial peptides; BAs, bile acids; FXR, farnesoid receptor; GPBAR1, G protein-coupled bile acid receptor 1; H2S, hydrogen sulfide; IFN-γ, interferon-γ; IL-10, anti-inflammatory factors; LPS, lipopolysaccharides; MMP-7, Matrix metalloproteinase-7; NF-κB, nuclear factor kappa B; NLRP3, nucleotide-binding and oligomerization domain-like receptor protein 3; ROS, reactive oxygen species; SCFAs, short-chain fatty acids; SR-A, type A scavenger receptor; TMAO, Trimethylamine N-oxide; Treg, regulatory T cells; TLR-7, Toll-like receptor-7; TH, T helper cell; TM, T memory cell.

The immune system is divided into innate and adaptive systems. Innate immunity includes physical barriers (such as the intestinal mucosa) and chemical barriers (such as various enzymes, antibacterial proteins, and immune cells). Adaptive immunity is divided into humoral and cellular immunity, which are mainly mediated by T and B cells, respectively, and are responsible for maintaining human health (Figure 1).

### Interplay between the gut microbiota and the innate immune system

#### Physical barriers

Innate immunity of the intestine is demonstrated in the process of direct contact between intestinal epithelial cells and the gut microbiota and its metabolites. Intestinal epithelial cells have two main functions, segregation and mediation (Okumura and Takeda, 2017). A tight junction between intestinal epithelial cells acts as a strong physical barrier to prevent bacteria from invading through the side of the cell, cup cells secrete a large amount of viscous fluid, forming a large reticulosyl protein mucin polymer that contains antibacterial molecules such as IgA and defensin family proteins, and mucus secretion is mainly regulated by the host’s perception of the microorganisms and their metabolites in the intestine (Marques and Boneca, 2011). The role of Paneth cells in the small intestine is more prominent because they contain antimicrobial peptides (AMPs) that play a key role in isolating intestinal bacteria and epithelium (Bevins and Salzman, 2011). AMPs are congenital immunoeffectors with bactericidal, anti-inflammatory, and anti-endotoxin properties that limit the interactions between pathogens and epithelial cells (Vandamme et al., 2012; Mergaert, 2018). The defensive elements of AMPs are divided into three categories: α, β, and θ, of which α-defensins mainly prevent pathogenic bacterial infections. Matrix metalloproteinase-7 (MMP-7) is essential for the maturation of α-defensins, and defective mice lacking MMP-7 are highly sensitive to Salmonella typhimurium (Wilson et al., 1999). Mediation refers to the transmission of information between cellular microorganisms and the host immune cells. The balance between host and gut microbes is guaranteed by the recognition of microorganisms by pattern recognition receptors (PRRs; Wiertsema et al., 2021), stimulated by intestinal microorganisms and their metabolites, intestinal epithelial cells activate PRRs to induce the production of cytokines and chemokines necessary for protective immune responses, induce T cell immune responses, or present antigens to antigen-presenting cells in lymphoid tissues, but achieve tolerance to specific IgA and food antigens (Fukata and Arditi, 2013). T cells are activated to release IL-17 and IL-22, thereby promoting the production of AMPs mediated by intestinal epithelial cells and regulating the overgrowth of pathogenic bacteria (Liang et al., 2006). Improper activation of PRRs can lead to excessive immune response, resulting in inflammation and autoimmune diseases. The expression of these substances can be enhanced by the presence of specific microorganisms. Therefore, the composition of the gut microbiota is an important factor that triggers an innate immune response (Iacob et al., 2018).

#### Macrophages

Resident macrophages in the gut are derived from human peripheral monocytes and provide the host with a stable and critical innate immune defense (Bain et al., 2012). Mature macrophages have phagocytic activity, manifested by the increased expression of antigen presentation of the major histocompatibility complex (MHC) class II molecule, presenting the antigen to T cells through lymphatic circulation, and its phagocytic activity is also associated with morphological changes, they can respond to intestinal bacterial pathogens by generating a 1.0 μm pore filter, sensing, capturing, and killing pathogens *via* appendages extending into the monolayer of the intestine, and mediating a significant pro-inflammatory microenvironment without destroying the epithelial barrier (Noel et al., 2017; Yang and Cong, 2021). Mononuclear cells infiltrate the mucosa in the plasma and differentiate into “reactive” macrophages, producing inflammatory mediators through TLR-7, while inducing neutrophil recruitment. For pathogenic factors (such as bacteria, viruses, and parasites), different macrophage populations can regulate the immune-inflammatory response of CD4+ T cells, such as Th1 and Th2, in the absence of lymphocytes; that is, macrophages may initiate and guide the T cell response (Mills et al., 2000; Mills and Ley, 2014). This is a condition for the formation of lipid streaks early in arteriosclerosis (Tabas and Bornfeldt, 2016), and the interaction between mucosal intestinal bacteria and the immune system damages cardiomyocytes increases the risk of heart failure (Sandek et al., 2012).

#### Dendritic cells

Dendritic cells (DCs) in the intestine are important mediators of antigen presentation and are an important link between innate and acquired immune systems with strong functional plasticity (Sun et al., 2020). The function of DCs is affected by various factors in the intestinal environment. SCFAs can promote the differentiation of classical DC type 2 while increasing the expression of retinaldehyde dehydrogenase (RALDH) enzymes in intestinal epithelial cells to promote mature regulatory T (Treg) cells, thus, inflammatory and pathological hypersensitivity reactions to harmful nutrients in the intestine are prevented (Scott et al., 2011; Tan et al., 2016; Goverse et al., 2017). DCs in the gut have superior T cell stimulation capacity compared to macrophages, both as effector cells to produce type I and III interferons (IFNs) and as excellent antigen-presenting cells (APCs; Pavli et al., 1993). Dietary salts are an important factor in DC activation, which induces T cells to produce the pro-hypertensive cytokines IL-17 and IFN-γ, resulting in salt-sensitive hypertension (Barbaro et al., 2017). In addition, activated DCs can be transformed from IFN-producing cells to classical APCs to stimulate naïve T cells and adaptive immune responses *in vivo*. The antigen molecule binds to MHC Class II when the exogenous antigen is absorbed by DCs and, in conjunction with the T cell co-stimulating molecule, initiates the intestinal drainage of naïve T cells in the lymph nodes (Lombardi and Khaiboullina, 2014). This process can exacerbate endothelial inflammation, leading to atherosclerosis progression (Gao et al., 2016).

#### Neutrophils

Non-specific defenses of neutrophils are important for maintaining homeostasis *in vivo*; however, their persistent activation and aggregation are common features of inflammatory diseases, neutrophils fight infections primarily by engulfing and degranulating the offending bacterial cells, when activated, they release neutrophil extracellular traps (NETs), which may drive inflammatory exacerbation, epithelial injury, and increased thrombotic tendency (Li et al., 2020), and may be involved in the formation of atherosclerotic plaques, arteriovenous thrombosis, and the development and progression of abdominal aortic aneurysms (Perez-Olivares and Soehnlein, 2021). When intestinal barrier function is disabled and bacterial components enter circulation, neutrophils are rapidly activated and exert an effect by inducing monocyte recruitment through the platelet cooperation mechanism. Furthermore, capturing pathogens can enhance the release and phagocytic activity of pro-inflammatory cytokines (Maugeri et al., 2014; Lin et al., 2020). The gut microbiota has a regulatory effect on neutrophil homeostasis, promoting vascular immune cell infiltration and inflammation driven by monocyte chemoattractant protein 1 (MCP-1)/IL-17, and promoting angiotensin II-induced vascular dysfunction and hypertension (Karbach et al., 2016). Specific gut microbiota induce serum amyloid production, which promotes Th-17 differentiation and plays a role in regulating the development and effect of neutrophils (Sano et al., 2016). Activated neutrophils persist in the circulation, and the neutrophil-lymphocyte ratio (NLR) is a readily available inflammatory biomarker, which may increase the risk of adverse cardiovascular events (Adamstein et al., 2021).

### Interplay between the gut microbiota and the adaptive immune system

#### T cells

Adaptive immunity, including cellular and humoral immunity, is the body’s specific immunity against various pathogens, they respond quickly and strongly to external antigens. CD4+ Treg cells expressing the transcription factor Foxp3 play a key role in limiting the inflammatory response in the intestine and are important in maintaining intestinal homeostasis (Campbell et al., 2020). Butyrate produced during fermentation by intestinal microorganisms and starch can promote the production of Treg cells [such as T helper 17 (Th17), Th1, and Th2] in the colon (Furusawa et al., 2013). Acetate and propionate promote the accumulation of Treg cells in the colon by activating GPR43, inducing and regulating Treg cell differentiation, and releasing IL-10 and transforming growth factor β (TGF-β) to prevent excessive inflammatory responses (Kim et al., 2013). The concentration of SCFAs in the lumen is positively correlated with the number of Tregs (Arpaia et al., 2013). Butyrate and propionate can block the production of dendritic cells by influencing specific transcription factors of dendritic cell precursors but do not affect granulocyte production (Singh et al., 2010). Therefore, SCFAs, metabolites of the gut microbiota, play an important anti-inflammatory role in maintaining intestinal immune homeostasis. Bile acid is a cholesterol-derived molecule that is converted into secondary bile acid metabolite 3-oxolithocholic acid (3-oxoLCA) through the action of microorganisms in the colon, controlling the differentiation of Th17 cells and reducing immune irritation (Paik et al., 2022). It can be summarized that metabolites produced from steroids under the action of the gut microbiota contribute to the immune balance of the intestine.

#### B cells

B cells, including T cell-dependent and non-dependent B cells, comprise the other part of intestinal adaptive immunity. T cell-dependent B cells mainly protect the host from microbial invasion by producing antibodies, such as IgA, other antibodies, including IgM and IgG, are mostly produced by T cell non-dependent B cells (Bunker et al., 2017; Zhao and Elson, 2018). Microbial antigens mainly activate B cells through B cell receptors, TLR, and other pathways, whereas microbial metabolites such as SCFAs can directly activate B cells and then differentiate into plasma cells that can secrete antibodies (Sanchez et al., 2020). The intestinal microbiota has a significant stimulatory effect on IgA production. For example, *fragile bacilli* in the intestinal tract are positively correlated with IgA production (Nakajima et al., 2020). In some cases, it may also have the opposite effect on homeostasis. Intestinal microbes promote the production of antibodies by B cells through activation of TLR, contributing to the progression of atherosclerotic disease (Chen et al., 2016). Although IgM and other antibodies have compensatory effects in the gut, IgA-deficient mice are prone to intestinal microecological disturbances that induce chronic intestinal inflammation, which also increases the risk of metabolic disease (Jorgensen et al., 2020). B cells control intestinal inflammation by releasing IL-10 (Rosser and Mauri, 2015). Moreover, memory B cells and long-lived plasma cells in the gastrointestinal tract provide persistent intestinal humoral immunity (Mesin et al., 2011).

### Metabolites

#### SCFAs

SCFAs, especially acetate, propionate, and butyrate, are the end products of dietary fibers and resistant starch after fermentation by the intestinal microbiota. *Bacteroides* spp. mainly produce acetate and propionate, whereas butyrate is mainly synthesized by Bacteroidetes and Clostridiales. SCFAs affect epithelial barrier defense function, regulate innate immune cells, and bidirectionally regulate adaptive immunity (Martin-Gallausiaux et al., 2021). First, SCFAs promote proliferation of intestinal epithelial cells, which strengthens the physical barrier function of the intestine and reduces the likelihood of intestinal bacterial invasion (Park et al., 2016). Second, SCFAs can induce directional migration (chemotaxis) of neutrophils to the foci of inflammation and destroy microbial pathogens, while specifically regulating the production of IL-10 and inhibiting LPS-induced pro-inflammatory factors, including nitric oxide (NO), IL-6, and IL-12 (Vinolo et al., 2011; Sun et al., 2018). Therefore, it alleviates chronic inflammation in the body. Thirdly, it can also upregulate the three important metabolic processes of glycolysis, oxidative phosphorylation, and fat metabolism in B cells, promote the differentiation of B cells into plasma B cells, and increase IgA and IgG expression (Kim et al., 2016). In addition, after binding to the free fatty acid receptors FFAR2 and FFAR3, SCFAs can boost the secretion of gut-derived satiety hormones to prevent the formation of fat cells owing to excessive energy intake (Larraufie et al., 2018).

Studies have shown that supplementation with a butyrate diet can inhibit the progression of atherosclerosis in ApoE knockout mice while reducing the production of chemotaxis protein 1 (CMP-1) at the site of vascular injury, vascular cell adhesion molecule-1(VCAM-1), and matrix metalloproteinase-2(MMP-2), reducing macrophage migration, and increasing collagen deposition and plaque stability (Kasahara et al., 2018). It is reasonable to assume that some SCFAs have a positive effect on the slowing of atherosclerosis. In heart failure, SCFAs induce NLRP3 inflammasome activation and the secretion of IL-18 in a GPR43- and GPR109A-dependent manner to maintain the integrity of the intestinal barrier (Venegas et al., 2019), Treg cell activation, proliferation, and release of anti-inflammatory factor IL-10, reducing the number of effector memory T cells and Th17 cells and the degree of myocardial damage (Sun et al., 2018). SCFAs, especially butyrate, are beneficial for hypoxia inducible factor 1α (HIF1α) and aryl hydrocarbon receptor (AhR) production, which can promote the expression of IL-22 in CD4 T cells, protect the intestines from inflammation, and maintain intestinal homeostasis (Marinelli et al., 2019; Yang et al., 2020).

#### TMAO

Trimethylamine N-oxide (TMAO) is an intestinal microbiome-dependent metabolite derived from dietary choline, L-carnitine, and betaine (Gatarek and Kaluzna-Czaplinska, 2021). The gut microbiota decomposes choline by promoting the division of carbon-nitrogen bonds, particularly by the phyla Firmicutes and Proteobacteria, and six microbial genera, such as *Anaerococcus hydrogenalis, Clostridium asparagiforme, Clostridium hathewayi, Clostridium sporogenes, Escherichia fergusonii, Proteus penneri, Providencia rettgeri*, and *Edwardsiella tarda.* Proteobacteria, Bacteroidetes, and Prevotellaceae are responsible for the degradation of L-carnitine (Romano et al., 2015). Eating a large amount of red meat or egg yolks promotes the production of trimethylamine (TMA) in the gut microbiota. TMA enters the circulatory system and is oxidized to trimethylamine N-oxide (TMAO) by hepatic flavin-containing monooxygenase (FMO), studies have shown that mice have reduced systemic TMAO levels when FMO3 is knocked out (Shih et al., 2019). Elevated levels of TMAO in the blood contribute to an increased incidence of atherosclerosis (Koeth et al., 2019; Zhu et al., 2020). By inducing CD36 and SR-A1 activation on the surface of macrophages, TMAO promotes the accumulation of cholesterol in cells while affecting the reverse transport of cholesterol. Lipid-overloaded macrophages become foam cells, leading to atherosclerosis (Wang et al., 2011). TMAO increases pro-inflammatory cytokines [TNF-α and interleukin-1β (IL-1β)] and reduces the production of IL-10 (Seldin et al., 2016), triggers NLRP3 activation and reactive oxygen species production, promotes vascular inflammation and endothelial damage, and enhances the expression of inflammatory genes by activating nuclear factor kappa B (NF-κB; Yang et al., 2019). Additionally, TMAO can increase platelet hyper-reactivity and thrombosis (Zhu et al., 2016). Together, these mechanisms promote atherosclerosis formation. Furthermore, the serum level of TMAO can predict clinical outcomes in patients with heart failure. TMAO levels are associated with elevated pulmonary artery and wedge pressure, which is an indicator of left atrial stress, and is associated with prognosis in patients with acute decompensated heart failure (Troseid et al., 2015).

#### Bile acids

Bile acids are the main components of bile and are important substances synthesized by the liver to digest cholesterol. Approximately 95% of bile acid is absorbed back into the liver through the ileum, and a small amount is excreted in the feces (Chiang and Ferrell, 2018). Cholesterol 7α-hydroxylase (CYP7A1) catalyzes the majority of bile acid synthesis in humans (>90%), and bile acid metabolism is regulated by two receptors: the nuclear farnesoid receptor (FXR) and the G protein-coupled bile acid receptor 1 (GPBAR1; Chiang and Ferrell, 2020). Among these, FXR is significantly activated at high serum cholesterol levels. Activated FXR maintains normal triacylglycerol (TAG) and cholesterol levels and regulates CVD risk factors such as lipid and glucose metabolism (Cariou and Staels, 2007). FXR activation can weaken the immune response and promote Treg cell differentiation. In the absence of FXR, the expression of inflammatory factors (such as IL-1β) in macrophages is decreased, and vascular damage is relieved (Kang et al., 2021). FXR also relies on toll-like receptor 9 (TLR9) to inhibit the pro-inflammatory response of intestinal macrophages. On the other hand, macrophages express high levels of GPBAR1, and activated GPBAR1 (also known as TGR5) can inhibit NF-κB to reduce the accumulation and activation of macrophages in aortic plaques and adipose tissue, and inhibit inflammation. Expression of such receptors is also found in other innate immune cells such as dendritic cells (Fiorucci and Distrutti, 2015; Fiorucci et al., 2018). Simultaneous inhibition of both receptors at the same time can block the anti-inflammatory effect and aggravate atherosclerosis (Miyazaki-Anzai et al., 2018). Bile acids also regulate the transcription of genes involved in low-density lipoprotein cholesterol (LDL-C) synthesis and cholesterol homeostasis *via* nuclear hormone receptors (Yamaoka-Tojo et al., 2008). This also increases the risk of atherosclerosis in patients with non-alcoholic fatty liver disease (Fiorucci and Distrutti, 2022).

#### H_2_S

Hydrogen sulfide (H_2_S) is a colorless, flammable, and water-soluble gas that is one of the richest metabolites in the gut microbiota. The production of endogenous hydrogen sulfide relies primarily on enzymatic production of cystathionine β synthase (CBS), cystionine-γ lyase (CSE), 3-mercaptopyruvate thiotransferase (3-MST), cysteine aminotransferase (CAT), and D-amino acid oxidase (DAO; Kimura, 2015; Panthi et al., 2016). The non-enzymatic pathway requires specific intestinal flora to ferment and degrade dietary inorganic polysulfides to produce H_2_S, the mechanism of different bacteria to produce H_2_S is different. *Bacillus anthracis, Pseudomonas aeruginosa,* and *Staphylococcus aureus* rely mainly on CBS and CSE to produce H_2_S, whereas *E. coli* does not contain CBS and CSE and relies mainly on the synergistic effect of CAT and 3-MST to produce H_2_S (Shatalin et al., 2011; Gui et al., 2021).

H_2_S is important for regulating macrophages and T cells, reducing oxidative stress, regulating vasomotor function, protecting the heart, and fighting atherosclerosis (Meng et al., 2015; Lv et al., 2021). Exogenous H_2_S supplementation or upregulation of CSE to promote endogenous H_2_S production inhibits NF-κB activation, enhances the DNA binding activity of the nuclear receptor (PPAR-γ), blocks fat cell formation, and inhibits the expression of macrophage chemokine receptors CX3CR1 to reduce macrophage chemotaxis (Zhang et al., 2012). H_2_S can also promote CD4 Foxp3 Treg cell differentiation, maintain H_2_S-NFYB (nuclear transcription factor Y subunit β)-Tet (Tet1 and Tet2 promoter) axis homeostasis, play an immunomodulatory role, reduce the inflammatory response of atherosclerotic plaques, and simultaneously enhance TCR-dependent T cell activation and IL-2 expression, which is considered to be a new immunomodulatory molecule for T cell response (Pan et al., 2017; Xiong et al., 2019). H_2_S can relax blood vessels by regulating calcium channels, reducing blood pressure, and constricting blood vessels after the concentration reaches a certain level, thus playing a regulatory role in maintaining blood pressure stability (Lv et al., 2021). Moreover, H_2_S can also block the RAS system, activate constitutional Ang II, improve Ang II-induced cell damage, and reduce the activation and oxidative stress of the NLRP3 inflammasome in spontaneously hypertensive mice (SHR; Li et al., 2019). H_2_S can attenuate nuclear factor erythroid 2-related factor 2/nuclear factor kappa B (Nrf2/NF-κB) signals, weaken oxidative stress, and inhibit mitochondrial ROS production to preserve mitochondrial function, decrease cardiac muscle infarct size, preserve cardio-contractile function, activate or rescue endothelial nitric oxide synthase (eNOS) functionality, and relieve heart failure (Testai et al., 2016; LaPenna et al., 2021; Ling et al., 2021).

## Role of the microbe-gut-heart-axis in the occurrence and development of CVDs

**Figure 2:**
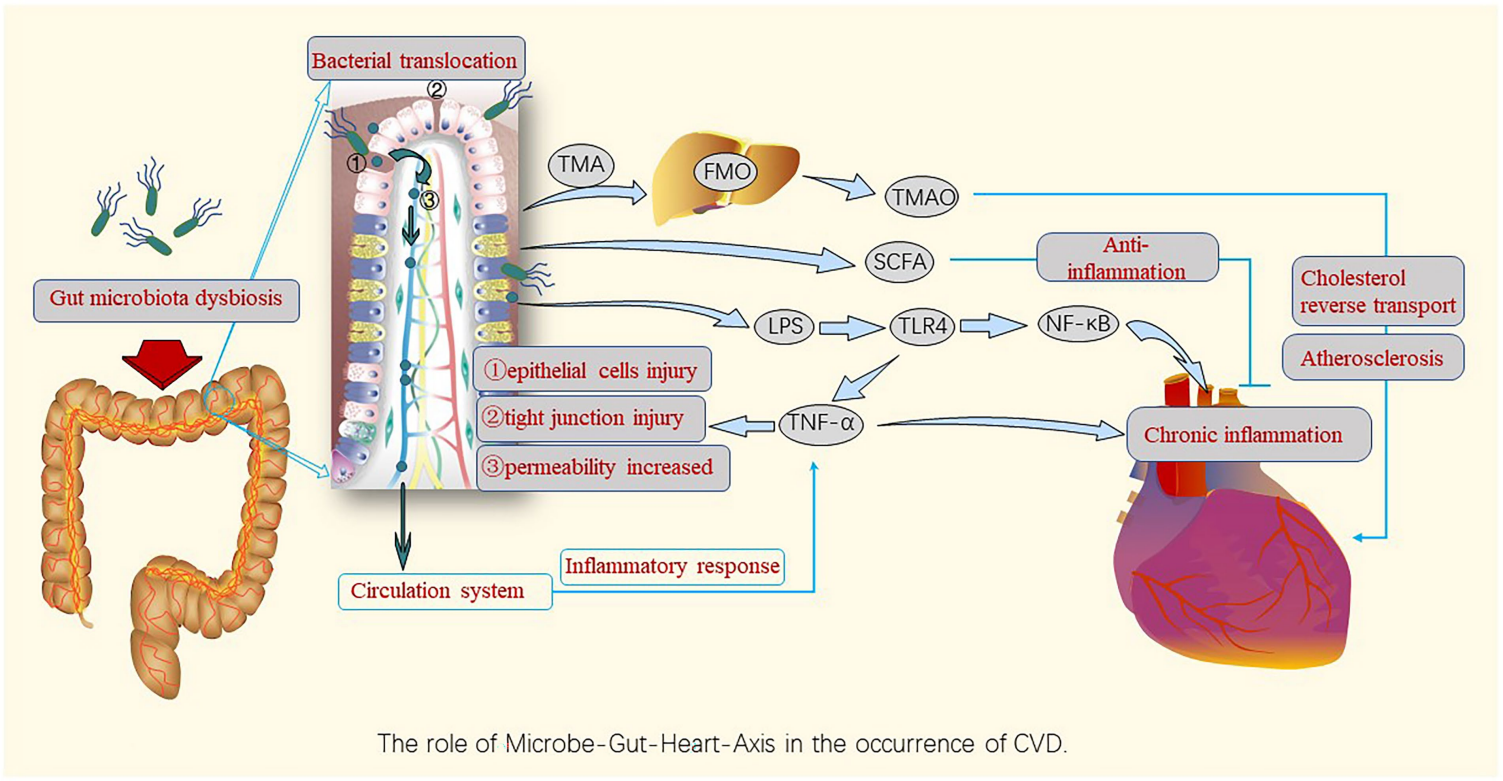
Roles of gut microbiota dysbiosis and bacterial translocation in the occurrence and development of CVDs. Gut microbiota dysbiosis destroys the barrier function, leading to gut bacteria leakage, entry of bacterial components into the circulatory system, and induction of chronic inflammation by harmful metabolites such as TMAO and pro-inflammatory factors, causing harm to the cardiovascular system *via* the gut-heart axis; however, SCFAs play an anti-inflammatory role in this axis. TMA, trimethylamine; FMO, flavin-containing monooxygenase; TMAO, Trimethylamine N-oxide; SCFAs, short-chain fatty acids; LPS, lipopolysaccharides; TLR-4,Toll-like receptor-4; NF-κB, nuclear factor kappa B; TNF, tumor necrosis factor.

The microbe-gut-heart-axis is a concept that illustrates the relationship between the intestinal microbiota and CVD, which has been confirmed by many studies. Figure 2 displays the role of the microbe-gut-heart-axis in the occurrence of CVDs. Gut dysbiosis causes inflammation and metabolic disorders. The F/B ratio is associated with maintaining homeostasis, and changes in this ratio can lead to various pathologies, such as hypertension, heart failure, obesity, and inflammatory bowel disease (Tang et al., 2019; Stojanov et al., 2020). In the state of the diseases, dysbiosis of the gut microbiota makes the intestinal barrier unstable, the intestinal permeability increases, and bacterial translocation occurs. This allows endotoxins and harmful metabolites to accumulate in circulation, causing chronic inflammation throughout the body and increasing the risk of CVD (Bartolomaeus et al., 2020). Thus, dysbiosis of the gut microbiota and bacterial translocation are the main mechanisms contributing to the imbalance between the gut microbiota and its metabolites and host immunity (Aaron et al., 2021; Ahlawat et al., 2021).

### Dysbiosis of the gut microbiota

Owing to the host’s diet, environment, mental factors, and antibiotics or other drugs, the gut microbiota and metabolites are mostly disordered during disease occurrence and development. Host diet is a crucial factor. A high-sodium diet is one of the most important risk factors for noncommunicable diseases worldwide (Collaborators, 2019). It can affect blood pressure and gut microbiota composition and function (Wang et al., 2017; Miranda et al., 2018). Additionally, it helps to replace beneficial bacteria and produce metabolites such as indole, which increases the incidence of cardiovascular events in patients with chronic kidney disease (Lin et al., 2012; Hung et al., 2017). As mentioned earlier, the gut microbiota has changed in patients with atherosclerosis (Mitra et al., 2015), hypertension (Li et al., 2017), and heart failure (Mamic et al., 2016), compared with healthy adults. This indicates that the homeostasis of the human internal environment in the disease state has a great influence on the gut microbiota, changing the normal ratio of good bacteria to bad bacteria in the gut. One study showed that *Lactobacillus rhamnosus* GG supplementation can efficiently reduce plasma TMAO levels in both humans and animals (Cantero et al., 2022), whereas another study showed that minocycline treatment induces a high F/B ratio and programmed hypertension, remodeling the gut microbiota with reduced *Lactobacillus*, *Ruminococcus*, and *Odoribacter* (Hsu et al., 2021).

### Bacterial translocation

Intestinal ischemic injury is a potential cause of leaky guts. Ischemia leads to alterations in intestinal epithelial cells and tight junctions, causing a defective intestinal barrier. This leads to increased permeability, allowing intestinal bacterial components to invade the circulatory system through paracellular transport and causing intestinal leakage (Nagpal and Yadav, 2017). Bacterial DNA have been detected in atherosclerotic plaques (Jie et al., 2017). Coronary atherosclerosis is a chronic inflammatory process, TNF-α induces intestinal epithelial barrier dysfunction, allowing LPS on the cell wall of gram-negative bacteria to leak into the circulation and bind to LPS-binding proteins, thus aggravating inflammation (Wang et al., 2012; Brandsma et al., 2019). Consequently, TLR4 is activated, and downstream inflammatory signal NK-κB conduction is initiated; NLRP3 inflammasomes are activated, promoting the overproduction of pro-inflammatory cytokines and adhesion molecules, activation of caspase 1, and secretion of IL-1β and IL-18, aggravating the atherosclerotic inflammatory response (Baldrighi et al., 2017). In addition, IL-17-producing Th17 cells maintain gut barrier integrity, and Th17 response failure contributes to increased gut permeability and favors the translocation of LPS into the circulatory system (Perez et al., 2019). Obesity (Genser et al., 2018), aging (Sharma, 2022), high-fat diet, high-intensity exercise (Aune et al., 2021), and autoimmune diseases may aggravate intestinal leakage. Moreover, patients with chronic heart failure have more intestinal leakage than healthy individuals, which increases the likelihood of inflammation spreading and accelerates the progression of CVD (Sandek et al., 2014; Pasini et al., 2016).

## Regulating the gut microbiota in CVDs

### Diet

Diet is an important regulator of the intestinal microbial composition and functional characteristics, which can change the composition of the gut microbiota (Lindskog Jonsson et al., 2018). Chronic low-grade inflammation caused by a high-fat diet can induce an increase in Enterobacter (phylum degenerate; Malesza et al., 2021). Polyphenols in fresh fruits and vegetables can be further metabolized in the large intestine, improving plasma HDL levels and reducing C-reactive protein and triglyceride levels. Additionally, they can have an inhibitory effect on pathogenic bacteria, such as *Clostridium capsulatum*. Although the vegan diet is rich in dietary fiber and polyphenols, it is not widely recommended because of the lack of important substances such as proteins and fats (Sakkas et al., 2020). As mentioned earlier, red meat consumption increases TMAO production, whereas long-term medical diet intervention can change the overall gut microbiota. For example, a Mediterranean diet will boost certain bacteria belonging to both Firmicutes and Bacteroidetes, inducing high production of SCFA (butyrate species), which is known as the best diet for preventing cardiovascular events. These have a strong protective effect on cardiovascular risk factors such as lipid metabolism and inflammation (Merra et al., 2020).

### Antibiotics

Antibiotics can cause a direct reduction in intestinal microbial diversity and even have adverse consequences, such as drug resistance reactions and *Clostridium difficile* infection (Ianiro et al., 2016). An increase in Enterobacteriaceae and other pathogens and a decrease in *Bifidobacterium* and butyrate-producing species were observed after the use of a cocktail of three last-resort antibiotics: meropenem, gentamicin, and vancomycin (Palleja et al., 2018). The host can also show different recovery times after the antibiotic destroys the microbiota: children have been reported to take approximately 1 month, whereas adults are mostly restored within 1.5 months (Ramirez et al., 2020; Yang et al., 2021; Duan et al., 2022). In the treatment of Crohn’s disease, the appropriate use of antibiotics reduces bacterial load, improves intestinal microecological disorders, and reduces abdominal pain, diarrhea, and other symptoms to a certain extent, however, this is limited to short-term treatment, and long-term use may contribute to disease progression (Lange et al., 2016). Therefore, to control antibiotic abuse, studies have focused towards antimicrobial peptides that can be combined with conventional antibiotics (Grassi et al., 2017). Nevertheless, nitrofurantoin treatment increased *Fecalibacterium* genus in some patients with urinary tract infections, and *Faecalibacterium is* a butyrate producer with strong anti-inflammatory properties, which provides a positive effect to CVDs (Stewardson et al., 2015). That means antibiotics are a potential treatment method to regulate the state of intestinal microecological disorders in patients with CVD.

### Probiotics and prebiotics

Probiotics are important for maintaining intestinal homeostasis and improving host intestinal health by interfering with pathogenic bacteria by competing for nutrition and specific adhesion sites, influencing immunomodulation, inhibiting pathogenic bacterial colonization, and maintaining intestinal barrier function (Butel, 2014; Wieers et al., 2020; Wang et al., 2021). Specific strains from *Lactobacillus*, *Bifidobacterium* can be used to reduce the risk factors of CVD, like obesity (Musazadeh et al., 2022). *Bifidobacterium* has long been considered a beneficial probiotic, and cross-feeding between *Faecalibacterium prausnitzii* and *Bifidobacterium adolescentis* can enhance butyrate formation (Rios-Covian et al., 2015). Individuals who received probiotic intervention (*L. rhamnosus* CNCM I-4036 or *L. paracasei* CNCM I-4034) showed a significant increase in the *Lactobacillus* and *Parabacteroides* genera and a decrease pro-inflammatory cytokines together with an increase in anti-inflammatory cytokines, which can confer health benefits to the host (Plaza-Diaz et al., 2015). Prebiotics, which are selectively fermented ingredients, allow specific beneficial changes in the composition and activity of gastrointestinal microbiota (Thomas et al., 2015). For example, fructo-oligosaccharides can stimulate the growth of probiotics, inhibit pathogen colonization, promote probiotic fermentation, digestion, and absorption of nutrients, stimulate anti-inflammatory cytokines to act on non-immune cells, and protect tissues from immunopathological damage (Zhou, 2017). Probiotics and prebiotics promote the recovery of intestinal ecological imbalances.

### Fecal microbiota transplantation

Fecal microbiota transplantation (FMT) is a direct method for regulating the host tract microbiota (Khanna et al., 2017). It originated in China in the fourth century AD and has since been widely used in veterinary medicine and in patients with gastrointestinal diseases, especially recurrent refractory *C. difficile* infection (Wang et al., 2019). A human study conducted by Vrieze et al. (2012) demonstrated that FMT could temporarily increase peripheral insulin sensitivity in patients with metabolic syndrome, which may be related to the high levels of butyrate-producing bacteria in the donor intestine (de Groot et al., 2017). FMT from donors of different body sizes shows varying degrees of insulin sensitivity in obese subjects with metabolic syndrome. This also provides ideas for the treatment of obesity, glucose metabolism, and CVD (Aron-Wisnewsky et al., 2019; Zoll et al., 2020). However, there are still some problems associated with this method, such as the risk of disease transmission, ambiguous impact on the recipient’s immune system, and secondary diseases (De Leon et al., 2013; Baxter and Colville, 2016). Additionally, living gut microbiota is highly sensitive to diet, drugs, and other factors, which cannot guarantee the long-term efficacy of microbiota transplantation. Therefore, researchers should further explore and study FMT.

## Conclusions and expectations

The relationship between CVDs and the gut microbiota and its metabolites has gradually been established. Different CVDs exhibit unique characteristics in the gut microbiota and their metabolites (Table 1) and are closely related to the host innate and adaptive immune systems (mainly through bacterial components and metabolites). Physical barriers are the first line of defense, whereas immune cells, (such as macrophages, dendritic cells, and neutrophils) interact with the gut microbiota and metabolites. This can have negative impacts and accelerate the progress of CVDs (Figure 1). The microbe-gut-heart-axis directly explains the relationship between the gut microbiota and CVDs through dysbiosis and bacterial translocation theories. Therefore, therapies such as diet adjustment, antibiotics, probiotics, and FMT from healthy volunteers are being used or will be used appropriately in clinical settings to alleviate the clinical symptoms and disease progression of patients with CVDs. Nevertheless, the detailed mechanisms of CVDs caused by some metabolites, such as TMAO, need to be studied in depth. Additionally, the interaction between the host immune system, intestinal bacteria, and metabolites is expected to become a new way to prevent and treat CVDs. The purpose of studying the immune mechanism between CVD and the gut microbiota and its metabolites is to provide a potential direction for various effective treatments of CVDs.

## Author contributions

MW designed and directed the manuscript. JL wrote the manuscript. SY and XJ revised the manuscript. YL and XW searched the literature databases. All authors contributed to the article and approved the submitted version.

## Funding

This work was supported by the National Natural Science Foundation of China (Grant Nos. 81202805 and 82074254), the Beijing Natural Science Foundation (No. 7172185) and the Science and Technology Innovation Project of the China Academy of Chinese Medical Sciences (No. C12021A01413).

## Conflict of interest

The authors declare that the research was conducted in the absence of any commercial or financial relationships that could be construed as a potential conflict of interest.

## Publisher’s note

All claims expressed in this article are solely those of the authors and do not necessarily represent those of their affiliated organizations, or those of the publisher, the editors and the reviewers. Any product that may be evaluated in this article, or claim that may be made by its manufacturer, is not guaranteed or endorsed by the publisher.
